# Invaginated Pancreaticojejunostomy via the Space Behind the Root of Superior Mesenteric Vessels

**DOI:** 10.4021/gr311e

**Published:** 2011-03-20

**Authors:** Fu Tian Du, Wei Ding, Hong Feng Lin, Xiao Xia Gong, Sen Li, Qin Hua Song

**Affiliations:** a Department of Hepatobiliary Surgery, Weifang People’s Hospital, Weifang 261041, China

**Keywords:** Retrospective study, Periampullary neoplasm, Surgery, Diagnosis, Pancreaticoduodenectomy, Pancreatic fistula

## Abstract

**Background:**

This study was to explore a safe and effective procedure to prevent pancreatic fistula (PF) after pancreaticoduodenectomy (PD).

**Methods:**

Forty-three modified PD with pancreaticojejunostomy by direct invagination of the pancreas to the jejunum that was brought up via the space behind the root of superior mesenteric vessel were performed between January 2003 and June 2006, and were compared to the fifty-six conventional PD (Child’ method).

**Results:**

There was no pancreatic fistula after PD in the modified group. Two cases developed biliary fistula that were successfully treated with complete drainage for 2 to 3 weeks; 2 cases abdominal infection managed with anti-infection and completely drainage; 4 cases stress ulcer cured with Losec and coagulant. Three cases in the Child group developed PF of different severities, with amylase level > 9000 U/L in the abdominal drainage fluid. Two of the PF were treated with Stilamin, parenteral nutrition, fasting and completely drainage and cured after 21 to 32 days. The other 82-year-old patient died. Other complications had no significant difference between the two groups (P > 0.05).

**Conclusions:**

The modified PD can effectively prevent PF and is a safe and effective procedure for periampullary neoplasm. Further studies of its clinical use are warranted.

## Introduction

Pancreaticoduodenectomy (PD) is the standard surgical method for periampullary neoplasm, which has been widely accepted worldwide. Pancreatic fistula (PF) is the most serious and commonest complication after PD with an incidence of about 13% and mortality rate up to 25% [1]. The prevention of PF remains a challenge for abdominal surgeons. We conducted a retrospective study of the patients at our hospital who underwent conventional pancreaticoduodenectomy (Child’s) or modified pancreaticoduodenectomy with pancreaticojejunostomy by direct invagination of the pancreatic stump into the jejunum which was brought up via the space behind the root of superior mesenteric vessel. There was no PF occurred in the modified group. The report is as follows.

## Patients and Methods

### Patient characteristics

From January 2003 to June 2006, 99 PD were performed at the Department of Hepatobiliary Surgery, Weifang People’s Hospital, Weifang, China. Patients were randomly selected for one of the two procedures, Child or modified PD.

Forty-three patients underwent modified PD (modified group), among them, 28 patients were male and 15 were female, patients were between 38 and 76 years of age (median 57). This group included 21 cases of carcinoma of the head and the uncinate process of pancreas, 11 cases of distal bile duct cancer, 6 cases of ampullary carcinoma, 2 cases of duodenal carcinoma, and 3 cases of duodenal papillary carcinoma.

The other 56 underwent the conventional Child PD (Child group), including 31 male and 25 female with ages between 28 and 82 years (median 55). This group included 1 case of cystadenoma of the pancreatic head, 27 cases of carcinoma of the head and uncinate process of pancreas, 14 cases of distal bile duct cancer, 7 cases of ampullary carcinoma, 5 cases of duodenal carcinoma, and 2 cases of duodenal papillary carcinoma. The preoperative blood test results for patients from both groups are presented in Table 1.

**Table 1:** Preoperative Blood Test for the 99 Patients Underwent Pancreaticoduodenectomy

Parameters	Modified group n = 43	Child group n = 56	t value	P value
ALT (IU/L)	231 ± 87	249 ± 78	1.082 4	> 0.05
AST (IU/L)	161 ± 79	210 ± 67	2.791 3	< 0.05
TBil (µmol/L)	213 ± 98	187 ± 101	1.286 0	> 0.05
TP (g/L)	76 ± 6.5	73 ± 5.4	2.507 1	< 0.05
A (g/L)	38.3 ± 3.4	36.8 ± 3.6	2.104 8	< 0.05
PT (s)	14 ± 1.8	13.6 ± 2.1	0.998 5	> 0.05
Hb (g/L)	123 ± 12	120 ± 14	1.123 3	> 0.05
BS (> 6.2 mmol/L)	8 cases	10 cases	0.03	> 0.05

### Operative technique

Duodenum and the head of pancreas were dissected and an exploration was carried out to detect evidence of infiltration and adhesion of pancreatic head to the inferior vena cava, and tumor invasion of the superior mesenteric vein (SMV), especially the uncinate tumor invasion of the right lateral side and the posterior side of the SMV, to assess the resectability of the tumor using conventional PD. In modified group, pancreas was transected on the left of SMV. One transverse suture was placed and tied at each of the superior and inferior edge of the pancreas proximal to the site of transection. Bleeding proximal to the transection edges were managed by ligation. All the sutures were left uncut to be used to draw the pancreas remnant into the jejunal lumen. The remnant pancreas was dissected free for 3 cm to ensure an invaginated anastomosis. A silastic tube can be inserted if the pancreatic duct was dilated.

**Figure 1:**
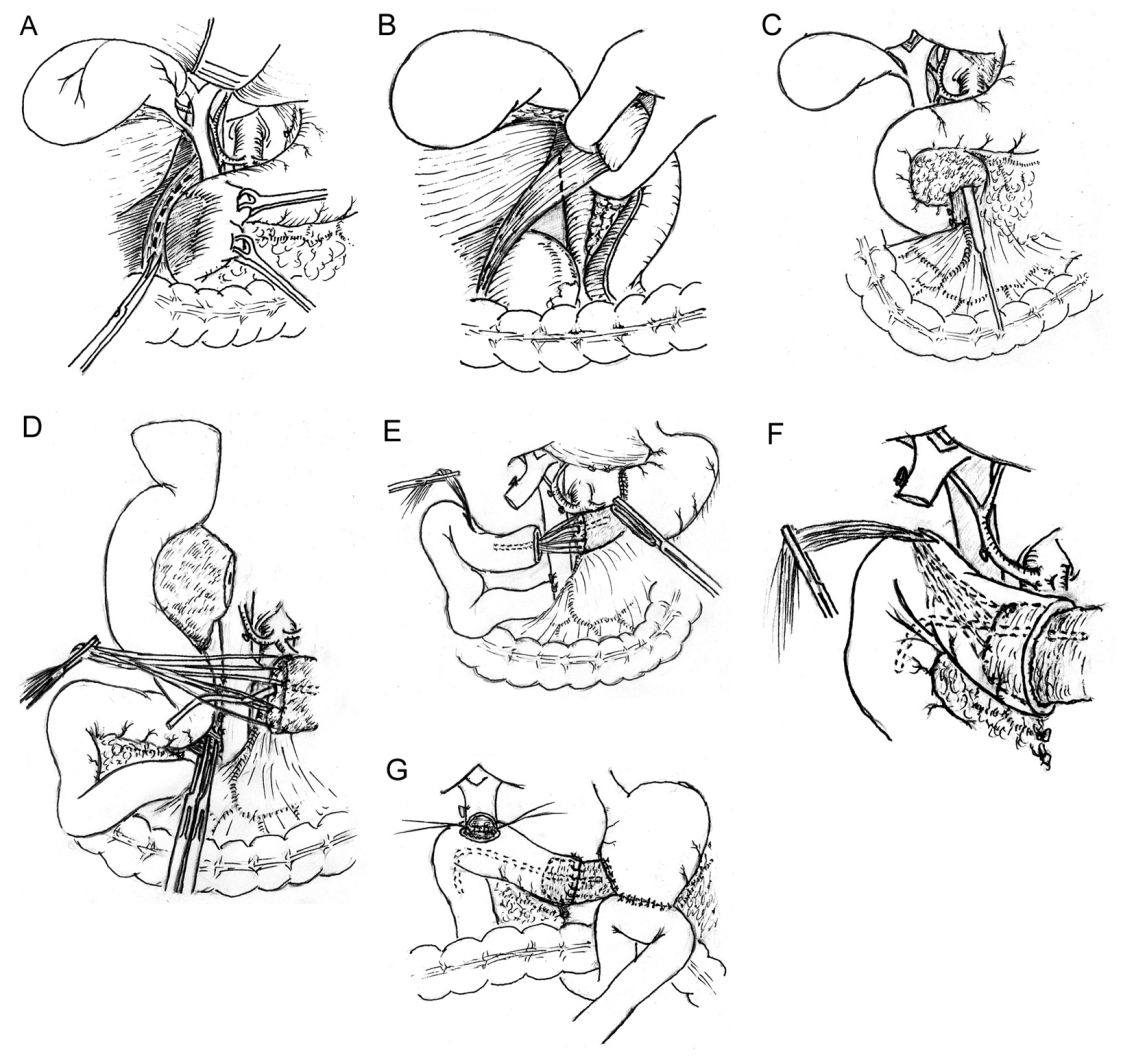
M*odified* pancreaticoduodenectomy with pancreaticojejunostomy by direct invagination of the pancreas into the jejunum that is brought up via the space behind the root of superior mesenteric vessel. (A, B) A *Kocher* maneuver is performed by incising the peritoneum lateral to the duodenum; the duodenum and the head of pancreas are reflected to the opposite direction; an inspection is carried out to detect if infiltration and adhesion of the tumor of pancreatic head to the inferior vena cava exists. (C) Using maxillary sinus stripper to explore evidence of infiltration and adhesion of tumor to the superior mesenteric *vein (SMV), especially* the uncinate tumor invasion of the right lateral surface and the undersurface of the SMV, *to assess the resectability of the tumor. (D)* All the sutures placed at the transected edges of the pancreas were left uncut to be used to pull the pancreas remnant into the jejunal lumen. The remnant pancreas was dissected free for 3 cm to ensure an invaginated anastomosis. The uncinate process of pancreas and the third and fourth portions of the duodenum were divided, leaving the jejunum intact. The upper jejunum wa*s mobilized after division of the ligament of Treitz and was then brought up through the space behind the root of* superior mesenteric vessels *to the region of pancreatic head and duodenum. The jejunum is transected approximately 10 cm from its beginning. (E, F) T*he end of the divided jejunum was drawn up to the pancreas remnant. A 1.5 cm enterotomy approximately 13 cm to the end of the transected jejunum is made on the antimesenteric surface of the jejunum with electrotome. *A large curved clamp is passed through the* enterotomy *and the sutures placed at the cut end of the pancreas are grasped and pulled out of the* enterotomy. Through pulling the sutures, the cut end of the pancreas is then invaginated 3 cm into the jejunal lumen; (G) Discontinuous sutures are placed circumferentially between the pancreas and the cut edge of the jejunum to complete the anastomosis.

The uncinate process of the pancreas and the third and fourth portions of the duodenum were divided, leaving the jejunum intact. The upper jejunum was mobilized after division of the ligament of Treitz and was brought up through the space behind the root of superior mesenteric vessels to the region of pancreatic head and duodenum. The jejunum was transected approximately 10 cm from its beginning. The isolated distal stomach, pancreatic head and uncinate process, duodenum, and proximal jejunum were removed. The end of the transected jejunum was then drawn up to the pancreas remnant. A 1.5 cm enterotomy approximately 13 cm to the end of the transected jejunum was made on the antimesenteric surface of the jejunum with electrotome, which would be used for cholangiojejunostomy. A large curved clamp was inserted into the enterotomy and the sutures at the cut end of the pancreas were grasped and pulled out of the enterotomy. Through pulling the sutures, the cut end of the pancreas was then invaginated 3 cm into the jejunal lumen, then discontinuous sutures were placed around the pancreas and the cut edge of the jejunum to complete the anastomosis. The cholangiojejunostomy and gastrojejunostomy were then performed. Double-cavity cannula was placed for irrigation and drainage. The abdominal wall was closed with a standard procedure. (Fig. 1).

### Statistical analysis

All analyses were conducted by statistical analysis software SPSS 13.0. Student’s t-test, χ^2^ test and rank sum test were used to compare the two groups. P values of less than or equal to 0.05 were considered statistically significant.

## Results

All the 43 modified PD were performed uneventfully. The mean duration of operation was 310 ± 38 min, compared with 320 ± 45 min for the conventional Child group. The mean operative blood loss was 550 ± 158 ml; it was 560 ± 145 ml for the Child group. Neither the duration of operation nor the operative blood loss was significantly different between the two groups (P > 0.05).

In the modified PD group, there was no PF after PD; biliary fistula occurred in 2 cases which were successfully treated with complete drainage for 2 to 3 weeks; 2 cases abdominal infection were managed with antibiotics and complete drainage; 4 cases of stress ulcer were cured with Losec and coagulant.

Three cases in the conventional Child group developed PF of different severities, with amylase level > 9000 U/L in the abdominal drainage fluid on or after day 3 postoperatively, and the total abdominal fluid drainage ranged between 300 and 1500 ml per 24 hours. Thus the incidence of PF in this group was 5.3%, significantly different from the modified group. Two of the PF were treated with Stilamin, parenteral nutrition, fasting and completely drainage and cured after 21 to 32 days. One case with previous ERBD died, during the operation the ERBD tube was found large and stiff, which compressed the opening of the pancreatic duct and caused severe pancreatic edema. Serious postoperative PF, infection and hemorrhage occurred, causing death on postoperative day 16. The incidence of other complications was not significantly different between the two groups (P > 0.05). A summary of the complications in the two groups was recorded in Table 2. The postoperative blood test results were presented in Table 3.

**Table 2:** Postoperative Complications in the 99 Patients Underwent Pancreaticoduodenectomy (%)

Complications	Modified group n = 43	Child group n = 56	χ^2^ value	P value
Total	11 (25.6)	14 (25)	0.004 4	> 0.05
Pancreatic fistula	0 (0)	3 (5.3)	2.375 6	< 0.05
Biliary fistula	2 (4.6)	3 (5.3)	0.025 3	> 0.05
Stress ulcer	4 (9.3)	3 (5.3)	0.576 2	> 0.05
Infection	2 (4.6)	3 (5.3)	0.025 3	> 0.05
Death	0 (0)	1 (1.8)	0.775 7	> 0.05
Delayed gastric emptying	3 (6.9)	2 (3.5)	0.588 2	> 0.05

**Table 3:** Postoperative Blood Test for the 99 Patients Underwent Pancreaticoduodenectomy

	Modified group n = 43	Child group n = 56	χ^2^ value	P value
Postoperative day 3				
ALT (IU/L)	212 ± 67	201 ± 71	0.782 9	> 0.05
AST (IU/L)	159 ± 76	196 ± 57	2.768 9	< 0.05
TBil (µmol/L)	163 ± 76	147 ± 89	0.943 7	> 0.05
Hb (g/L)	117.7 ± 17.6	111.9 ± 16.4	1.689 6	> 0.05
A (g/L)	33.4 ± 4.1	32.2 ± 4.6	30.627 9	< 0.05
AMS (IU/L)	167 - 236	173 - 9,762	0.032 4	< 0.05
Postoperative day 7				
ALT (IU/L)	167 ± 46	159 ± 53	0.787 7	> 0.05
AST (IU/L)	137 ± 61	143 ± 59	0.494 2	> 0.05
TBil (µmol/L)	67 ± 21.3	63 ± 28.6	0.767 8	> 0.05
Hb (g/L)	116.9 ± 15.9	116.8 ± 15.7	0.031 2	> 0.05
A (g/L)	35.2 ± 4.1	34.3 ± 5.9	0.854 0	> 0.05
AMS (IU/L)	51 - 107	46 - 10,716	0.027 6	< 0.05

## Discussion

In addition to the common complications after abdominal surgery, the early postoperative complications after PD mainly include PF, biliary fistula, hemorrhage, stress ulcer, delayed gastric emptying, and so on. Among which PF is the most serious and commonest one [2-4]. Activated amylase in the pancreatic fluid leakage erodes into and digests adjacent tissues including blood vessels, resulting in potentially lethal intra-abdominal hemorrhage. To prevent PF, more than 30 various pancreatic anastomotic reconstruction methods have been described, mainly including end-to-end anastomosis with invagination of the pancreatic stump in inverted jejunum, duct-to-mucosa pancreaticojejunostomy, pancreaticogastrostomy, pancreatic duct embolization, duct ligation, etc. Pancreatic duct ligation is a simple procedure, but the postoperative PF incidence can reach 50%, thus it is not in use anymore. The current PF incidence ranges between 5 - 10%. Although there are many different anastomotic methods [5, 6], none of them could eliminate the occurrence of PF. A report by the Massachusetts General Hospital demonstrated an incidence of 9.6% of PF in 733 patients underwent PD in the recent 10 years [7].

In an attempt to prevent PF based on the Peng’s binding pancreaticojejunostomy, we have developed a new technique for pancreaticojejunostomy, which consists of the direct invagination of the pancreatic stump into the jejunum that is brought up via the space behind the root of superior mesenteric vessel [8-10]. This method is easy, simple and time saving, and reduces the incidence of PF due to the complete invagination of the pancreatic stump into the jejunal lumen. The key points of this method include: 1) the remnant pancreas was dissected free for 3 cm to ensure an invaginated anastomosis. Care must be taken not to injure the splenic vein; 2) All the suture placed at the transected edges of the pancreas are left uncut to be used to pull the pancreas remnant into the jejunal lumen; 3) After the complete invagination of the pancreatic stump into the jejunal lumen, discontinuous sutures are placed around pancreas and the cut edge of the jejunum to complete the anastomosis; 4) In Peng’s binding pancreaticojejunostomy, the jejunal mucosa is destroyed using electrocautery or carbolic acid to induce the adhesion between the jejunum and pancreas. However, animal experiments demonstrate that it is not necessary to destroy the mucosa. To omit this step will shorten operative time and prevent edema of the jejunum; 5) It is not necessary trying to find the opening of the transected pancreatic duct, no matter it is dilated or not. When pancreatic duct dilation occurs, a silastic tube can be inserted to drain the pancreatic secretions into the jejunal lumen.

In the conventional PD, before incision of the uncinate process of pancreas and duodenum, the jejunum is transected under the colon. The cut end of the jejunum is pulled out behind the root of vessels and brought up through the mesenteric incision, then Child’s or other types of reconstruction is performed. In this modality, the space previously occupied by the pancreatic head and duodenum will be filled, however, a dead space remains behind of the root of vessels which is hard to be completely drained, resulting in fluid accumulation and affects healing of the pancreaticojejunostomy and hepaticojejunostomy.

In our modified method, jejunum is brought up to the pancreatic remnant through the space behind the root of superior mesenteric vessels, a course similar to the anatomical arrangement of duodenum, thus filling both the space formerly occupied by the pancreatic head and the one behind the root of vessels. It reduces the opportunities of fluid accumulation and infection, thus is helpful in the anastomotic healing.

In conclusion, the results demonstrate that our modified pancreaticojejunostomy, by direct invagination of the pancreas to the jejunum that is brought up via the space behind the root of superior mesenteric vessel, is simple, easy and time saving. It can prevent the postoperative PF after PD, and improve the safety of the operation.
